# Mid-term outcomes of atrioventricular valve repair in functional single ventricle patients

**DOI:** 10.3389/fcvm.2024.1510143

**Published:** 2024-12-18

**Authors:** Yong-Qiang Jin, Qing-Yu Wu, Xiao-Ya Zhang, Li-Xin Fan, En-Rui Zhang, Hui Xue, Ming-Kui Zhang

**Affiliations:** ^1^ Heart Center, The First Hospital of Tsinghua University, Beijing, China; ^2^ School of Clinical Medicine, Tsinghua University, Beijing, China

**Keywords:** single-ventricle, atrioventricular valve repair, Fontan, Glenn, operation timing

## Abstract

**Background:**

Surgical treatment of functional single ventricle combined with atrioventricular valve regurgitation remains a clinical challenge. The outcomes of atrioventricular valve repair in patients with single ventricle are limited.

**Methods:**

A retrospective study was conducted of all 28 patients with functional single ventricle treated with single-ventricle palliation who underwent atrioventricular valve operation at the First Hospital of Tsinghua University between April 2007 and October 2022.

**Results:**

In our cohort, the female/male ratio was 7:21, with an average age of 8.7 ± 6.0 (0.75–26) years. Half of patients (50%) were right-ventricle type for single-ventricle morphology. 18 patients (64.3%) were with a common atrioventricular valve. Twenty-three patients (82.1%) were combined with heterotaxy syndrome. Pre-operatively, twenty-four patients (85.7%) were diagnosed with severe atrioventricular valve regurgitation. AVV was repaired at the Glenn (*n* = 16, 57.1%), Glenn-Fontan (*n* = 2, 7.1%) and Fontan (*n* = 10, 35.7%) stage, respectively. Valve plastic techniques included valve annulus/commissure constriction (*n* = 24), clefts repair (9 cases), edge-to-edge suturing (13 cases) and common atrioventricular valve separation (4 cases). The early mortality was 3.6% (1/28). All survival patients were observed with improved regurgitation situations. Twenty-two patients (78.5%) were observed with no more than mild regurgitation postoperatively. The mean follow-up time was 5.4 ± 2.9 years (range, 3.08–11.83 years), with late mortality of 11.1% (3/27). All these three cases were observed with a severe regurgitation by echocardiogram in the last follow-up. Besides, reoperation rate of this cohort was 3.6% (1/28).

**Conclusions:**

AVV repair could significantly improve AVV function in SV patients combined with severe AVVR, with satisfactory mid-term results. Part of the cohort showed poor prognosis due to repeated AVVR. Regular follow-up by echocardiogram is critically important for these patients.

## Introduction

Since the Fontan procedure was first employed in humans in 1971, the outcomes for patients with functional single-ventricle heart disease (FSV) have significantly advanced (1). However, several factors continue to influence the efficacy of treatment and prognosis for FSV. Among these, atrioventricular valve regurgitation (AVVR) stands out as one of the most critical determinants (2, 3). Numerous studies have reported that moderate to severe AVVR serves as an independent risk factor for increased mortality and a higher incidence of complications (1–5).

Given the complexity of anatomical abnormalities and physiological challenges associated with FSV, managing coexisting AVVR remains a significant clinical challenge for congenital cardiac surgeons. Moreover, there is ongoing debate regarding indications for surgery, optimal timing of intervention, and appropriate techniques for valve repair. Consequently, strategic and efficient management of patients suffering from FSV conditions complicated by AVVR has consistently represented a major concern requiring careful attention within clinical practice. To address this issue, we conducted a single-institution retrospective cohort study involving 28 consecutive cases of FSV combined with AVVR who underwent atrioventricular valve repair surgery between April 2007 and October 2022. This study aims to review our surgical experiences with this unique patient population.

## Methods

### Ethical approval

Our study received approval from the Committee and Institutional Review Board of The First Hospital of Tsinghua University (Beijing Hua-Xin Hospital), Beijing, China. All procedures associated with this study, including the collection of medical records and follow-ups, were conducted in accordance with the Declaration of Helsinki. Given the retrospective nature of this research, the requirement for written informed consent was waived.

### Patient selection and data collection

Between April 2007 and October 2022, a total of 28 patients with FSV combined with moderate to severe AVVR receiving atrioventricular valve repair surgery at the Heart Center, The First Hospital of Tsinghua University were consecutively enrolled in this cohort study. We systematically collected their medical records, including demographic information (age, gender, race, weight, height, etc.), narrative history, electrocardiography (ECG, Mindray, Beneheart-R), chest x-ray, echocardiography (Echo, GE VIVID E95), chest computed tomography (CT), cardiac catheterization imaging, operative details and post-operative data for subsequent analysis. When patients were discharged from our hospital, we further collected follow-up information through clinical consultations and regular telephone interviews.

### Evaluation of atrioventricular valve regurgitation

We evaluated the extent of atrioventricular valve regurgitation (AVVR) primarily through routine transthoracic echocardiographic (TTE) examinations conducted at various time points: pre-operatively, post-operatively, prior to discharge, and during follow-up. Both two-dimensional echocardiography and color Doppler techniques were employed for this assessment. The severity of AVVR was classified into the following categories: none (0), trivial (1), mild (2), moderate (3), and severe (4).

### Statistical methods

There were no instances of drop-out or withdrawal during the follow-up period, resulting in a 100% follow-up rate among patients in our study. Statistical analyses were performed using SPSS software (version 22.0, IBM Corp., Armonk, NY). Normal data are presented as mean ± standard deviation or as percentages, while non-normally distributed data (categorical variables) are reported as median and range. Survival analysis was conducted utilizing the Kaplan-Meier method.

## Results

### Patient characteristics

A total of 28 consecutive patients with functional single ventricle (FSV) and atrioventricular valve regurgitation (AVVR) were enrolled in this study. Among the participants, there were 21 males and 7 females. The mean age of these patients was 8.7 ± 6.0 years, with a range from 8 months to 26 years. The average weight recorded for these patients was 25.9 ± 15.1 kg, spanning from 9.6 kg to 59.5 kg.

As summarized in Table 1, the primary diagnoses included: unbalanced atrioventricular septal defect (AVSD) in 18 cases (64.2%), mitral atresia in 2 cases (7.1%), tricuspid atresia in 3 cases (10.7%), double inlet ventricle in 4 cases (14.3%), and double outlet right ventricle in one case (3.6%). Regarding FSV morphology, there were classified as follows: Right-Ventricle Type accounted for 14 cases (50%), Left-Ventricle Type represented by six cases (21.4%), and Double-Ventricle Type comprising eight cases (28.6%).

**Table 1:** Preoperative information of all included 28 FSV patients.

Baseline characteristics		
Age (Mean ± SD, Range)		8.7 ± 6.0 (0.75–26)
Weight (Mean ± SD, Range)		25.9 ± 15.1 (9.6–59.5)
Gender *N* (%)	Female	7 **(**25%)
	Male	21 **(**75%)
Primary diagnosis *N* (%)	Unbalanced AVSD	18 (64.2%）
	Mitral atresia	2 (7.1%）
	Tricuspid atresia	3 **(**10.7%)
	Double inlet ventricle	4 **(**14.3%)
	Double outlet right ventricle	1 **(**3.6%)
SV morphology *N* (%)	Right-ventricle Type	14 **(**50%)
	Left-ventricle Type	6 **(**21.4%)
	Double-ventricle Type	8 **(**28.6%)
AVV morphology *N* (%)	Common AVV	18 **(**64.3%)
	Single-mitral valve	3 **(**10.7%)
	Single-tricuspid valve	2 **(**7.1%)
	Bilateral atrioventricular valve	5 **(**17.9%)
Other malformations *N* (%)	Right-atrium heterotaxy	15 **(**53.6%)
	Left-atrium heterotaxy	2 **(**7.1%)
	Situs inversus	6 **(**21.4%)
	Asplenia	16 **(**57.1%)
	Poly-splenia	4 **(**14.3%)
AVVR grade	Severe	24 **(**85.7%)
	Moderate	4 **(**14.3%)

Data are presented as mean ± SD or median for continuous variables or numbers and percentages for categorical variables.

SV, single ventricle; AVV, atrioventricular valve; AVVR, atrioventricular valve regurgitation; UAVSD, unbalanced atrioventricular septal defect.

For atrioventricular valve morphology, findings indicated that common atrioventricular valve (CAVV) was present in 18 cases (64.3%), while single mitral morphology was observed in three cases (10.7%) and single tricuspid morphology appeared in two cases (7%). Additionally, five instances of bilateral atrioventricular valves accounted for 17.9%.

In terms of other anatomical malformations, heterotaxy syndrome was identified among23 individuals (82.1%); specifically, this included 15 cases of right atrial isomerism, two of left atrial isomerism, and six of situs inversus. Furthermore, there were 16 cases of asplenia (57.1%) and four cases of polysplenia (14.3%).

### Operative details

There were 14 patients (50%) receiving previous cardiac palliations. The mean duration from the initial operation to the present operation was 5.7 ± 3.2 years, ranging from 2.5 to 12 years. Previous procedures included Blalock-Taussig procedure (3 cases, 10.7%), bidirectional Glenn procedure (11 cases, 39.2%). Among the 11 cases with previously bidirectional Glenn operations, 2 cases also underwent combined AVV repair surgery in other hospitals.

The operative details for all these including 28 patients were shown in Table 2. All these patients received operations under a general anesthesia (GA) and with cardiopulmonary bypass (CPB). The mean CPB time was 184.2 ± 81.6 h (58–422 h). The mean aortic cross-clamp (ACC) time was 75.1 ± 41.8 h (24–169 h). All patients (28/28, 100%) received scheduled repair surgeries. Among these patients, mostly (27/28, 96.4%) received AVV repair operations under cardiac arrest. Another patient underwent a beating-heart surgery via the left intercostal thoracotomy approach. The timing of AVV repair surgery included Glenn (16 cases, 57.1%), Glenn-Fontan (2 cases, 7.1%) and Fontan (10 cases, 35.7%) stage, respectively.

**Table 2:** Timing of atrioventricular valve surgery and detailed operative techniques.

Operative details	Procedures	*N* (%)
CPB time		184.18 ± 81.63 (58–422)
Aortic cross clamp		75.07 ± 41.84 (24–169)
Timing of surgery *N* (%)	Glenn	16 (57.1%)
	Glenn-Fontan	2 (7.1%)
	Fontan	10 (35.7%)
Repair techniques *N* (%)	Valve anulus/commissure constriction	24 (85.7%)
	Valve cleft repair	9 (32.1%)
	Edge-to-edge suture	13 (46.4%)
	CAVV separation	4 (14.3%)
Other combined operation *N* (%)	TAPVC corrections	6 (21.4%)
	PA constrictions	4 (14.3%)
	ASD enlargements	1 (3.6%)
	Glenn anastomotic stenosis correction	1 (3.6%)

Data are presented as numbers and percentages for categorical variables.

CAVV, common atrioventricular valve; TAPVC, total anomalous pulmonary venous drainage; PA, pulmonary artery; ASD, atrial septal defect; CPB, cardiopulmonary bypass.

Based on the diverse variations of structural anomalies or lesions associated with AVV morphology, a range of corresponding valve repair techniques were employed. These included constrictions of the valve annulus and/or commissures (24 cases, 85.7%), repairs of valve clefts (9 cases, 32.1%), edge-to-edge suturing methods (13 cases, 46.4%), and separations of CAVV (4 cases, 14.3%).

Apart from the Glenn or Fontan procedures, additional combined interventions were performed to address anatomical corrections for total anomalous pulmonary venous drainage (TAPVC) in 6 cases (21.4%), pulmonary artery (PA) constrictions in 4 cases (14.3%), atrial septal defect (ASD) enlargements in 1 case (3.6%), and correction of Glenn anastomotic stenosis in 1 case (3.6%). During these operations, all patients underwent transesophageal echocardiography (TEE) examinations to assess the effectiveness of atrioventricular valve repair and evaluate cardiac function.

### Early outcomes

All patients underwent routine follow-up after discharge. Patients who received the Fontan procedure are required to take lifelong aspirin medication at a dosage of 3–5 mg/kg/day. The mean duration of mechanical ventilation was 60.2 ± 115.0 h, with a range from 3.0 to 600.0 h. The average length of stay in the ICU was 10.3 ± 9.0 days, ranging from 1.0 to 36.0 days. The overall average length of hospital stay (LOS) was 29.6 ± 13.5 days, with a range from 9.0 to 54.0 days.

There was one case of mortality during the early postoperative period, resulting in an early mortality rate of 3.6%. The cause of death was attributed to low cardiac output syndrome (LCOS), which occurred on the fourth day following surgery.

### Detailed complications

As summarized in Table 3, the observed complications during the early postoperative phase included low cardiac output syndrome (LCOS) in 2 cases (7.1%), acute kidney injury (AKI) in 3 cases (10.7%), arrhythmia also in 3 cases (10.7%), and extracorporeal membrane oxygenation (ECMO) implantation in 2 cases (7.1%). Of the three patients who experienced arrhythmias postoperatively, one was diagnosed with sick sinus syndrome (SSS) and subsequently underwent permanent pacemaker implantation (PPI). Notably, all ECMO implantations occurred in patients undergoing Fontan surgery combined with atrioventricular valve repair; both of these patients had their ECMO withdrawn coincidentally on the fourth day after surgery and were discharged without any adverse events (AEs).

**Table 3:** Details of complications at early phase after operation.

Complications	*N* (%)
LCOS	2 (7.1%)
AKI	3 (10.7%)
Arrhythmias	3 (10.7%)
PM	1 (3.6%)
ECMO	2 (7.1%)

Data are presented as numbers and percentages for categorical variables.

LCOS, low cardiac output syndrome; AKI, acute kidney injury; SSS, sick sinus syndrome; ECMO, extracorporeal membrane oxygenation.

### Regurgitations

As presented in Table 4, all patients demonstrated an improvement in their aortic valve regurgitation (AVVR) status. Specifically, there were 2 cases classified as having no to trace regurgitations (Grade 0–1: 7.1%), 20 cases with mild regurgitations (Grade 2: 71.4%), and 5 cases exhibiting moderate regurgitation (Grade 3: 17.9%).

**Table 4:** Regurgitations after operation.

Regurgitations	*N* (%)
No-to-trace (Grade 0–1)	2 (7.1%)
Mild (Grade 2)	20 (71.4%)
Moderate (Grade 3)	5 (17.9%)

Data are presented as numbers and percentages for categorical variables.

### Follow-up

There were no drop-outs or withdrawals during the follow-up period. The mean follow-up duration was 5.4 ± 2.9 years, with a range of 3.08 to 11.83 years. During the follow-up, three patients died, resulting in a crude long-term mortality rate of 11.1% (3/27). The causes of mortality for these three patients included malignant arrhythmia (1 case), acute cerebral infarction (1 case), and acute heart failure (1 case). Additionally, all three patients exhibited severe regurgitation during the follow-up period.

Furthermore, one patient underwent reoperation for repair in the sixth year following the initial procedure; thus, the crude reoperation rate within our reported cohort was calculated to be 3.7% (1/27). Among the remaining 24 survivors, eleven individuals (39.3%) achieved Fontan completion.

## Discussion

The treatment of FSV remains a clinical challenge, especially when it occurs in combination with significant AVVR. In this present retrospective cohort report, we reviewed our single-centered clinical experiences for the surgical treatment of FSV combined with AVVR in 28 consecutive pediatric patients. It showed a satisfactory result with varying valve repair techniques. The early and long-term mortality rate and reoperation rate are relatively low in this cohort. Our results hint that the elimination of regurgitation may have close relationship with the prognosis for these FSV patients, especially for the mortality, as we observed all these three patients who died during the follow-up were found a severe recurrent regurgitation with TTE examinations.

Functional single ventricle (FSV) is one of the most complex CHD, in which clinical outcomes are markedly improved with the wide application of the Fontan procedure (6–8). However, when it is complicated with significant atrioventricular valve regurgitation, it is always correlated with a poorer outcome. Meanwhile, it is common in FSV patients complicated with AVVR, based on our literature review, the incidence of AVVR in FSV patients were 10.0%–13.5% (9, 10). Extensive literatures were conducted to explore the mechanisms of atrioventricular regurgitation in FSV patients, from animal studies, and bench to clinical studies (11–14). The mechanism of atrioventricular regurgitation in FSV patients is often the structural abnormality of atrioventricular valve apparatus, including valve leaflets, valve sinus, annulus, chordae tendineae and papillary muscles (15). When regurgitation occurs in younger children, especially in newborns, or in hypoplastic left heart syndrome (HIHS) patients, it is always related to a worst outcome (16). Intervention for regurgitation in the early phase may benefit to cardiac function and clinical outcomes. Honjo et al. reported that bi-directional Glenn procedure may not alleviate the progression of atrioventricular regurgitation, because the chronic pre-load increase may further enlarge the univentricular chamber and lead to the enlargement of the atrioventricular valve annulus, finally aggravate the regurgitation and impair cardiac function (5). Thus, it is critically important to the timing of intervention based on an accurate evaluation of the degree of regurgitation.

In this report, the timing of intervention for regurgitation varies according to the patient's disease characteristics and cardiac function. The timing of repair surgery included Glenn Stage (16 cases, 57.1%), Glenn-Fontan Stage (2 cases, 7.1%) and Fontan Stage (10 cases, 35.7%), respectively. All these repair operations were performed in patients with moderate or severe (Grade 3–4) regurgitations. According to our experience, if an FSV patient was observed with no more than Grade 2 (trace or mild) regurgitation, a regular follow-up is necessary without reintervention. However, if the FSV patients were observed with moderate or severe regurgitations, an aggressive anti-heart failure therapy should be conducted. If moderate to severe AVVR existed after treatment, timely valve repair or replacement operation is imperative. Otherwise, the cardiac function will be further impaired, and when it is extremely severe atrioventricular regurgitation with severe cardiac function impairment, only cardiac transplantation works (5, 15). Therefore, the timing of intervention for regurgitation may be closely related to the outcomes of these patients.

In this study, we reported varying repair techniques including valve annulus and/or commissure constrictions (24 cases, 85.7%), valve clefts repairs (9 cases, 32.1%), edge-to-edge suturing (13 cases, 46.4%) and CAVV separations (4 cases, 14.3%), according to the structural lesions of atrioventricular valve apparatus. Thus, it is also important to choose the appropriate repair techniques, which may also be related to the operative outcomes of these patients. Based on previous literatures, the essential principle in treatment of AVVR is, when it is can be repaired, valve repair procedure is the best choice than replacement. However, when the patient is in an older age, with extensive enlarged valve annulus or enlarged univentricular chamber, the quality of leaflets is poor, or extremely difficult to repair, replacement surgery may be the better choice. Overall, the technique selection should be individualized and comprehensive, fully considering the actual situation of the patient's characteristics (17, 18).

As our experience paid more attention on the timing of intervention and applications of varying repair techniques for AVVR, our results were satisfactory. We conducted a literature review for published cohorts reporting treatment of SV with AVVR, as shown in Table 5, our report seems to achieve a good surgical result, with achievement of most superior hospitalization mortality rate (3.6%) and considerably acceptable mid-term mortality rate (11.1%) (4, 9, 10, 19, 20). Meanwhile, the reoperation rate in our cohort was the lowest compared with other reported studies. However, there were still one case died at early phase post-operatively, as well as three cases died during the follow-up time. The critical question for the reported mortality was whether these patients would benefit more from AVVR replacement surgery compared with AVVR repaired. The question will be confirmed through further comparable and larger sample size, prospective studies. In addition, the ICU stay time and LOC time seemed a little longer in our cohort, which reflected the severity of illness for these AVVR repaired patients. Part of these patients also had to undergo ECMO treatment. However, through a good heart team, involving cardiac surgeons, ICU specialists, ECMO specialists, cardiologist and nursing team, we achieved a rather good result in these critical illness patients. In addition, we acknowledge that the Glenn operation, particularly when performed alongside concomitant valvular interventions, is considered safe based on several literature reports. However, a contentious debate continues regarding whether the Fontan procedure and valvular intervention should be conducted simultaneously. Some scholars advocate for a staged surgical approach as potentially safer. Nevertheless, drawing from our center's extensive experience, we assert that judiciously selecting appropriate candidates for concurrent Fontan and valvular interventions can be both safe and reliable.

**Table 5:** Literature review of SV combined with AVVR cohorts.

Publications	Publication time	Sample size	AVVR intervention rate	Early mortality rate	Mid-term mortality	2+ regurgitation	Reoperation rate	Repair/replacement ratio
Our study	NA	28	100%	3.6%	11.1%	21.5%	3.6%	28/0
Mayr et al. (19)	2021	66	45.5% (30/66)	23.3%	8.7%	33.8%	68.9%	28/2
Nakata et al. (20)	2010	65	100%	4.6%	30%	61.0%	49%	65/0
Misumi et al. (4)	2014	38	100%	13.2%	30%	NA	15%	38/0
Sinha et al. (9)	2021	60	100%	6.7%	13.3%	30%	18%	60/0
Honjo et al. (10)	2011	57	100%	14%	31.3%	14%	17%	57/0

Besides, based on our experience, the threshold for considering a repair unsuccessful in this specific cohort of patients, thereby justifying conversion to valve replacement, is defined as moderate to severe valve regurgitation observed postoperatively. In our cohort, no significant regurgitations were noted among the three late deaths. However, during the follow-up period, all these patients who experienced late death exhibited severe regurgitation without any intervention. Therefore, we suggest establishing moderate to severe valve regurgitation as the threshold for deeming a repair unsuccessful and necessitating conversion to replacement.

The present study has several limitations. Firstly, the study design is a retrospective, and single-institution cohort study, a relatively small sample size may affect its validation in other real-world settings. Secondly, the mean follow-up duration of this study is 5.4 ± 2.9 years, which reported the midterm outcomes of these patients, further longer follow-up data should be cumulated in order to assess the long-term safety and clinical effect of AVVR repair surgery. Last but not least, due to the relatively small sample size, no complicated statistical methods were applied in this present study, such as multivariate analysis or Cox-regression model. We expect to collect more patients' data to evaluate the risk factors that affect the mortality, reoperation rate or Fontan completion rate in further larger sample size, multi-centered studies.

In conclusion, our reports showed a satisfactory result for FSV patients combined with moderate to severe AVVR. Postoperative regurgitation may significantly affect the prognosis, especially the mortality. Routine echocardiogram follow-up postoperatively is critically important for these patients.

## Data Availability

The original contributions presented in the study are included in the article, further inquiries can be directed to the corresponding author/s.

## References

[1] King G Ayer J Celermajer D Zentner D Justo R Disney P Atrioventricular valve failure in Fontan palliation. J Am Coll Cardiol. (2019) 73(7):810–22. 10.1016/j.jacc.2018.12.025 30784675

[2] Tseng SY Siddiqui S Di Maria MV Hill GD Lubert AM Kutty S Atrioventricular valve regurgitation in single ventricle heart disease: a common problem associated with progressive deterioration and mortality. J Am Heart Assoc. (2020) 9(11):e015737. 10.1161/JAHA.119.015737 32419552 PMC7429008

[3] Naito Y Hiramatsu T Kurosawa H Agematsu K Sasoh M Nakanishi T Long-term results of modified Fontan operation for single-ventricle patients associated with atrioventricular valve regurgitation. Ann Thorac Surg. (2013) 96(1):211–8. 10.1016/j.athoracsur.2013.02.029 23623547

[4] Misumi Y Hoashi T Kagisaki K Kitano M Kurosaki K Shiraishi I Long-term outcomes of common atrioventricular valve plasty in patients with functional single ventricle. Interact Cardiovasc Thorac Surg. (2014) 18(3):259–65. 10.1093/icvts/ivt508 24336698 PMC3930217

[5] Honjo O Mertens L Van Arsdell GS. Atrioventricular valve repair in patients with single-ventricle physiology: mechanisms, techniques of repair, and clinical outcomes. Semin Thorac Cardiovasc Surg Pediatr Card Surg Annu. (2011) 14(1):75–84. 10.1053/j.pcsu.2011.02.001 21444052

[6] Hasaniya NW Razzouk AJ Mulla NF Larsen RL Bailey LL. *In situ* pericardial extracardiac lateral tunnel Fontan operation: fifteen year experience. J Thorac Cardiovasc Surg. (2010) 140(5):1076–83. 10.1016/j.jtcvs.2010.07.068 20951258

[7] Giannico S Hammad F Amodeo A Michielon G Drago F Turchetta A Clinical outcome of 193 extracardiac Fontan patients: the first 15 years. J Am Coll Cardiol. (2006) 47(10):2065–73. 10.1016/j.jacc.2005.12.065 16697327

[8] Kim SJ Kim WH Lim HG Lee JY. Outcome of 200 patients after an extracardiac Fontan procedure. J Thorac Cardiovasc Surg. (2008) 136(1):108–16. 10.1016/j.jtcvs.2007.12.032 18603062

[9] Sinha R Altin HF McCracken C Well A Rosenblum J Kanter K Effect of atrioventricular valve repair on multistage palliation results of single-ventricle defects. Ann Thorac Surg. (2021) 111(2):662–70. 10.1016/j.athoracsur.2020.03.126 32454017

[10] Honjo O Atlin CR Mertens L Al-Radi OO Redington AN Caldarone CA Atrioventricular valve repair in patients with functional single-ventricle physiology: impact of ventricular and valve function and morphology on survival and reintervention. J Thorac Cardiovasc Surg. (2011) 142(2):326–35.e2. 10.1016/j.jtcvs.2010.11.060 21592529

[11] De Silva M Tagliavia C Galiazzo G Gifuni G Caiazza M Chiocchetti R Morphological variability of the atrioventricular valve cusps in the equine heart. Equine Vet J. (2022) 54(1):167–75. 10.1111/evj.13434 33555625

[12] Rhinehart JD Schober KE Scansen BA Yildiz V Bonagura JD. Effect of body position, exercise, and sedation on estimation of pulmonary artery pressure in dogs with degenerative atrioventricular valve disease. J Vet Intern Med. (2017) 31(6):1611–21. 10.1111/jvim.14814 28865107 PMC5697194

[13] Takemura N Koyama H Motoyoshi S. Congestive heart failure due to bilateral atrioventricular valve insufficiency in two dogs. J Vet Med Sci. (1996) 58(4):381–4. 10.1292/jvms.58.381 8741276

[14] Jang WS Kim WH Choi K Lee JR Kim YJ Kwon BS What factors predict long-term survival and valve durability in patients with atrioventricular valve regurgitation in single-ventricle physiology? Pediatr Cardiol. (2013) 34(6):1366–73. 10.1007/s00246-013-0650-3 23397336

[15] Emani SM. Single ventricle with atrioventricular valve regurgitation-ongoing challenges. Ann Thorac Surg. (2021) 111(2):670–1. 10.1016/j.athoracsur.2020.04.059 32504608

[16] Pigula FA Mettler B. Management of tricuspid regurgitation in patients with hypoplastic left heart syndrome. Semin Thorac Cardiovasc Surg. (2017) 29(1):64–9. 10.1053/j.semtcvs.2017.02.004 28684000

[17] Mavroudis C Stewart RD Backer CL Deal BJ Young L Franklin WH. Atrioventricular valve procedures with repeat Fontan operations: influence of valve pathology, ventricular function, and arrhythmias on outcome. Ann Thorac Surg. (2005) 80(1):29–36; discussion 36. 10.1016/j.athoracsur.2005.01.071 15975335

[18] Sano S Fujii Y Arai S Kasahara S Tateishi A. Atrioventricular valve repair for patient with heterotaxy syndrome and a functional single ventricle. Semin Thorac Cardiovasc Surg Pediatr Card Surg Annu. (2012) 15(1):88–95. 10.1053/j.pcsu.2012.01.014 22424513

[19] Mayr B Burri M Strbad M Cleuziou J Hager A Ewert P Common atrioventricular valve surgery in children with functional single ventricle. Eur J Cardiothorac Surg. (2021) 60(6):1419–27. 10.1093/ejcts/ezab220 34008032

[20] Nakata T Fujimoto Y Hirose K Tosaka Y Ide Y Tachi M Atrioventricular valve repair in patients with functional single ventricle. J Thorac Cardiovasc Surg. (2010) 140(3):514–21. 10.1016/j.jtcvs.2010.05.024 20584537

